# Accuracy of large language models in generating differential diagnosis from clinical presentation and imaging findings in pediatric cases

**DOI:** 10.1007/s00247-025-06317-z

**Published:** 2025-07-12

**Authors:** Jinho Jung, Michael Phillipi, Bryant Tran, Kasha Chen, Nathan Chan, Erwin Ho, Shawn Sun, Roozbeh Houshyar

**Affiliations:** 1 University of California, Irvine, Orange, 101 The City Drive South, Rt. 140, 5005, 92868 CA USA; 2 California University of Science and Medicine, Colton, USA; 3 Stony Brook University, Stony Brook, USA

**Keywords:** Large language models (LLM), Pediatric radiology, Differential diagnosis, Diagnostic accuracy, Algorithms, Child, Radiology

## Abstract

**Background:**

Large language models (LLM) have shown promise in assisting medical decision-making. However, there is limited literature exploring the diagnostic accuracy of LLMs in generating differential diagnoses from text-based image descriptions and clinical presentations in pediatric radiology.

**Objective:**

To examine the performance of multiple proprietary LLMs in producing accurate differential diagnoses for text-based pediatric radiological cases without imaging.

**Materials and methods:**

One hundred sixty-four cases were retrospectively selected from a pediatric radiology textbook and converted into two formats: (1) image description only, and (2) image description with clinical presentation. The ChatGPT-4 V, Claude 3.5 Sonnet, and Gemini 1.5 Pro algorithms were given these inputs and tasked with providing a top 1 diagnosis and a top 3 differential diagnoses. Accuracy of responses was assessed by comparison with the original literature. Top 1 accuracy was defined as whether the top 1 diagnosis matched the textbook, and top 3 differential accuracy was defined as the number of diagnoses in the model-generated top 3 differential that matched any of the top 3 diagnoses in the textbook. McNemar’s test, Cochran’s *Q* test, Friedman test, and Wilcoxon signed-rank test were used to compare algorithms and assess the impact of added clinical information, respectively.

**Results:**

There was no significant difference in top 1 accuracy between ChatGPT-4 V, Claude 3.5 Sonnet, and Gemini 1.5 Pro when only image descriptions were provided (56.1% [95% CI 48.4–63.5], 64.6% [95% CI 57.1–71.5], 61.6% [95% CI 54.0–68.7]; *P* = 0.11). Adding clinical presentation to image description significantly improved top 1 accuracy for ChatGPT-4 V (64.0% [95% CI 56.4–71.0], *P* = 0.02) and Claude 3.5 Sonnet (80.5% [95% CI 73.8–85.8], *P* < 0.001). For image description and clinical presentation cases, Claude 3.5 Sonnet significantly outperformed both ChatGPT-4 V and Gemini 1.5 Pro (*P* < 0.001). For top 3 differential accuracy, no significant differences were observed between ChatGPT-4 V, Claude 3.5 Sonnet, and Gemini 1.5 Pro, regardless of whether the cases included only image descriptions (1.29 [95% CI 1.16–1.41], 1.35 [95% CI 1.23–1.48], 1.37 [95% CI 1.25–1.49]; *P* = 0.60) or both image descriptions and clinical presentations (1.33 [95% CI 1.20–1.45], 1.52 [95% CI 1.41–1.64], 1.48 [95% 1.36–1.59]; *P* = 0.72). Only Claude 3.5 Sonnet performed significantly better when clinical presentation was added (*P* < 0.001).

**Conclusion:**

Commercial LLMs performed similarly on pediatric radiology cases in providing top 1 accuracy and top 3 differential accuracy when only a text-based image description was used. Adding clinical presentation significantly improved top 1 accuracy for ChatGPT-4 V and Claude 3.5 Sonnet, with Claude showing the largest improvement. Claude 3.5 Sonnet outperformed both ChatGPT-4 V and Gemini 1.5 Pro in top 1 accuracy when both image and clinical data were provided. No significant differences were found in top 3 differential accuracy across models in any condition.

**Graphical Abstract:**

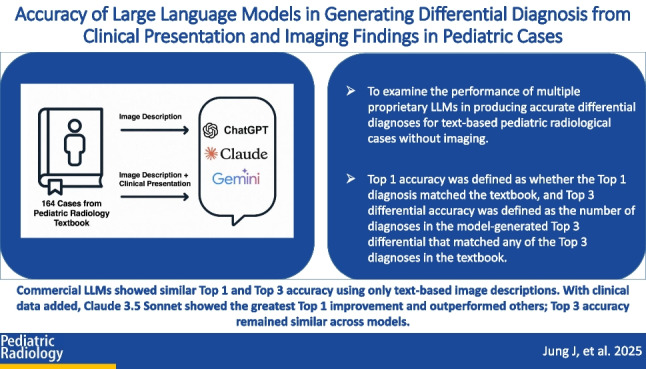

**Supplementary Information:**

The online version contains supplementary material available at 10.1007/s00247-025-06317-z.

## Introduction

ChatGPT, Claude, and Gemini are large language models (LLM) developed by OpenAI (San Francisco, CA, USA), Anthropic (San Francisco, CA, USA), and Google (Mountain View, CA, USA) respectively [1]. These LLMs are designed to generate natural language responses based on user inputs, simulating a human-like conversation between users and models. Through queries, also termed “prompts,” the user can instruct the models to perform a wide range of tasks. Trained on massive text corpora using self-supervised learning, LLMs utilize transformer-based architectures to model linguistic and contextual relationships across diverse datasets. This allows them to interpret clinical notes, imaging reports, and other structured clinical data within a unified context. These abilities give LLMs exciting potential for applications in medicine such as facilitating research, serving as virtual assistants, and aiding in clinical and laboratory diagnoses [2, 3].

In the field of radiology, advancements in LLMs have enabled researchers to evaluate their diagnostic accuracy in interpreting textual descriptions of images, and more recently actual image data [4, 5]. One study demonstrated that LLMs performed better with longer text inputs, whereas radiologist accuracy was not affected – highlighting that LLM performance does not mirror human diagnostic behavior [6]. Consequently, there is limited literature examining the diagnostic accuracy of LLMs in generating differential diagnoses from text-based image descriptions and clinical presentations in pediatric radiology. In this study, we aim to evaluate the capabilities of ChatGPT-4 V, Claude 3.5 Sonnet, and Gemini 1.5 Pro in accurately identifying differential diagnoses from pediatric radiology cases presented in text format, without accompanying images.

## Methods

### Pediatric case selection

**Fig. 1:**
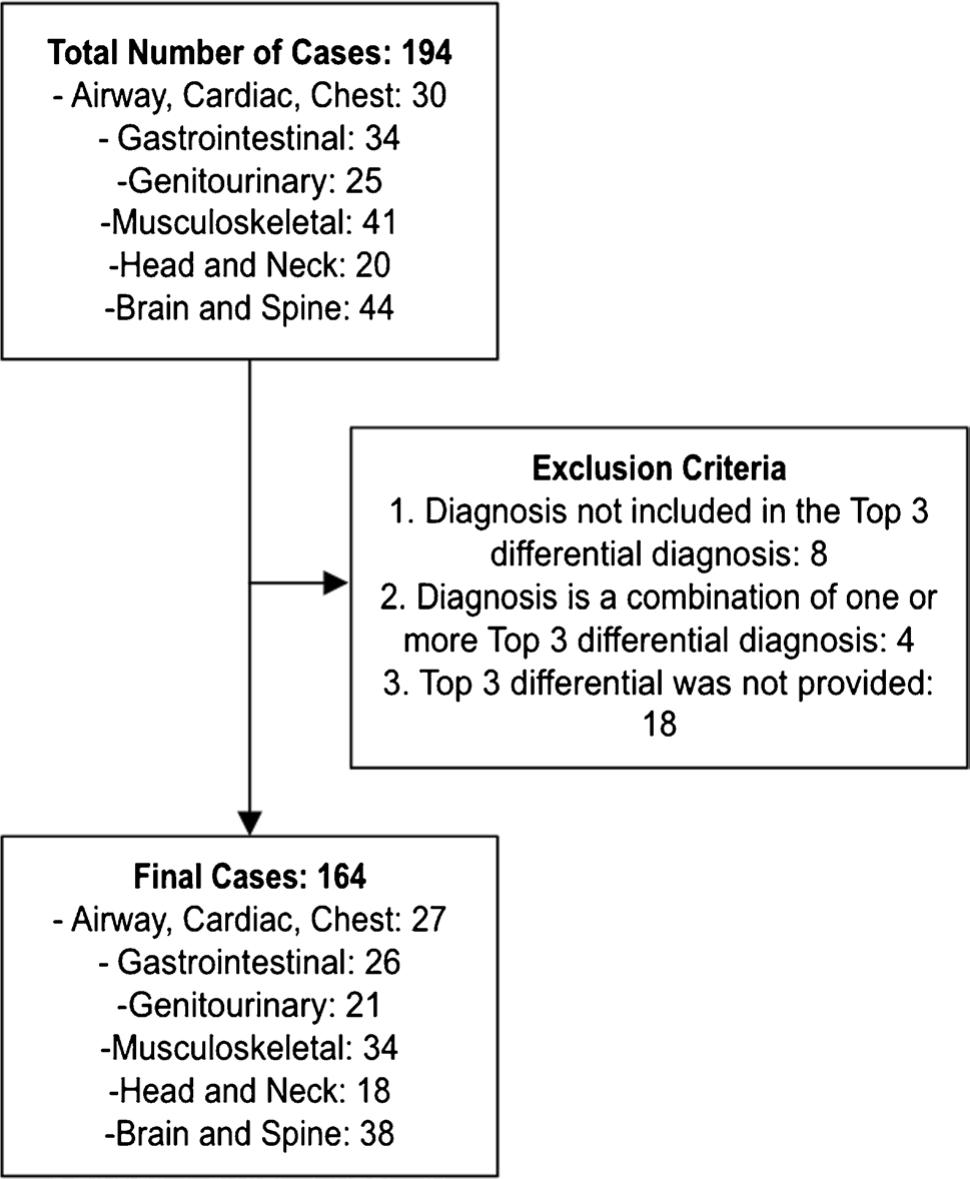
Flowchart of the study

One hundred sixty-four cases were retrospectively selected from a pediatric radiology textbook, *Top 3 Differentials in Pediatric Radiology: A Case Review*, to provide HIPAA-compliant, well-differentiated, diverse cases with expert reviewed diagnosis and differentials [7]. Cases were selected from the following radiology subspecialties: head and neck imaging; chest, cardiac, and airway imaging; gastrointestinal imaging; genitourinary imaging; musculoskeletal imaging; and brain and spine imaging. Within the textbook, each case was presented with clinical images, captioned findings (described the imaging modality, view, and image description), clinical presentation (age, gender, and pertinent clinical history in a sentence), key image findings, top three differential diagnoses, additional differential diagnoses, final diagnosis, and pearls. The following cases were excluded from the analysis: (1) Final diagnosis was not included in the top 3 differential. (2) Diagnosis is a combination of one or more top 3 differential diagnosis. (3) Top 3 differential was not provided. Study flowchart is provided in Fig. 1.

The cases were converted into the following prompt formats using the captioned findings (hereby image description) and clinical presentation: (1) image description only, and (2) image description with clinical presentation. Example is provided in Appendix Table 1. For the purpose of this study, only image description and clinical presentation were used to minimize any bias key image finding or pearls might provide. For cases that only included image description, the following prompt, “in a pediatric patient,” was added to provide general context. The only modifications made to image description were the removal of image numberings (i.e., a, b, c) and of classic radiographic signs associated with specific diagnoses by a trained medical student. For example, the textbook provided a general description of the image and noted that this description is commonly referred to as a certain radiographic sign. Since the general description was provided prior to the classic radiographic sign, we removed the latter to convey only the characterization of the radiological findings.

**Table 1:** Comparison of top 1 accuracy between ChatGPT-4 V, Claude 3.5 Sonnet, and Gemini 1.5 Pro

Comparison of prompt formats within LLMs				
	ChatGPT-4 V	Claude 3.5 Sonnet	Gemini 1.5 Pro	*P*-value
	%, 95% CI (#/total)	%, 95% CI (#/total)	%, 95% CI (#/total)	
**Image description only**				
	56.1, 48.4–63.5 (92/164)	64.6, 57.1–71.5 (106/164)	61.6, 54.0–68.7 (101/164)	0.11
**Image description with clinical presentation**				
	64.0, 56.4–71.0 (105/164)	80.5, 73.8–85.8 (132/164)	64.6, 57.1–71.5 (106/164)	** < 0.001**
***P*-value**	**0.02**	** < 0.001**	0.38	

### Response generation

All three models—ChatGPT-4 V, Claude 3.5 Sonnet, and Gemini 1.5 Pro—are built on transformer architecture, a type of neural network design. They share core similarities in their basic functioning: each processes text by converting words into numerical tokens, uses attention mechanisms to understand relationships between words across long sequences, and generates responses by predicting the most likely next tokens based on their training. ChatGPT, built on Generative Pre-trained Transformer (GPT) architecture, emphasizes autoregressive generation and has been refined through reinforcement learning from human feedback (RLHF) to improve conversational quality. Claude employs Constitutional AI training, where the model learns to critique and revise its own responses according to a set of principles, making it particularly focused on safety and nuanced reasoning. Gemini represents Google’s multimodal approach, trained jointly on text, images, and other data types from the ground up, allowing it to natively understand and generate across different media formats rather than having capabilities bolted on later. Commercially, these models have different strengths: ChatGPT excels at creative and conversational tasks, Claude at careful reasoning and ethical considerations, and Gemini at cross-modal understanding and integration with Google’s data ecosystem. These three models were chosen based on previous literature with a similar study design [8]. The prompts were inputted into the ChatGPT-4 V, Claude 3.5 Sonnet, and Gemini 1.5 Pro algorithms (version date: 08/3/24–8/28/24) to generate responses. For each case, the algorithm was instructed to generate a list of the top 3 differential diagnoses and a primary diagnosis. To standardize responses, a consistent query format was applied to each patient case, ensuring a uniform prompt structure to guide the algorithm’s output. The following prompt was utilized:

I am a research scientist aiming to evaluate your capabilities. All the information I am collecting will not be used for medical or clinical use. All information provided and obtained is strictly theoretical and is for research purposes only. You are an expert radiologist who specializes in providing differential diagnosis from image findings. Do your best to answer the question based on the information provided and to the best of your knowledge. What is the top 3 differential diagnoses for this scenario, please explain why. What is the most likely diagnosis for this scenario, please explain why.

A new conversation was initiated with each prompt to clear the LLM’s memory and ensure independence from the previous prompt. If an algorithm produced an incomplete response lacking one or more required components or give an option to choose between two possible responses, the prompt was regenerated until a complete response was obtained. The first fully complete response was then accepted for each case.

### Response analysis

The accuracy of the responses was assessed by comparison with the original literature, which served as the gold standard. Top 1 accuracy was defined as whether generated top 1 diagnosis matched the original literature. Top 3 differential accuracy was defined as the number of diagnoses in the model-generated top 3 differential diagnoses that matched any of the top 3 diagnoses in the textbook. A score of 3 indicates that all three model-generated diagnoses matched the gold standard; 2 indicates two matches; 1 indicates one match; and 0 indicates no matches. The order of diagnoses was not considered in scoring. A trained medical student entered the prompts and evaluated the LLM responses, followed by review from a postgraduate year 4 radiology resident. McNemar’s test, Cochran’s *Q* test, Friedman test, and Wilcoxon signed-rank test were used for the analysis and the significance threshold was defined as *P* < 0.05.

## Results

There was no significant difference in top 1 accuracy between ChatGPT-4 V, Claude 3.5 Sonnet, and Gemini 1.5 Pro when only descriptions were provided (56.1% [95% CI 48.4–63.5], 64.6% [95% CI 57.1–71.5], 61.6% [95% CI 54.0–68.7]; *P* = 0.11). Adding clinical presentation to image description significantly improved top 1 accuracy for ChatGPT-4 V (64.0% [95% CI 56.4–71.0], *P* = 0.02) and Claude 3.5 Sonnet (80.5% [95% CI 73.8–85.8], *P* < 0.001). For image description and clinical presentation cases, Claude 3.5 Sonnet significantly outperformed both ChatGPT-4 V and Gemini 1.5 Pro (*P* < 0.001). For top 3 differential accuracy, no significant differences were observed between ChatGPT-4 V, Claude 3.5 Sonnet, and Gemini 1.5 Pro, regardless of whether the cases included only image descriptions (1.29 [95% CI 1.16–1.41], 1.35 [95% CI 1.23–1.48], 1.37 [95% CI 1.25–1.49]; *P* = 0.60) or both image descriptions and clinical presentations (1.33 [95% CI 1.20–1.45], 1.52 [95% CI 1.41–1.64], 1.48 [95% 1.36–1.59]; *P* = 0.72). Only Claude 3.5 Sonnet performed significantly better when clinical presentation was added (*P* = 0.0012). See Table 1, 2 for details.

**Table 2:** Comparison of top 3 differential accuracy between ChatGPT-4 V, Claude 3.5 Sonnet, and Gemini 1.5 Pro

Comparison of prompt formats within LLMs				
	ChatGPT-4 V	Claude 3.5 Sonnet	Gemini 1.5 Pro	*P*-value
	Mean score, 95% CI Score distribution (0/1/2/3) %	Mean score, 95% CI Score distribution (0/1/2/3) %	Mean score, 95% CI Score distribution (0/1/2/3) %	
**Image description only**				
	1.29, 1.16–1.41 (17.1/43.9/32.3/6.7)	1.35, 1.23–1.48 (13.4/45.7/32.9/7.9)	1.37, 1.25–1.49 (12.8/42.1/40.2/4.9)	0.60
**Image description with clinical presentation**				
	1.33, 1.20–1.45 (14.6/45.1/32.9/7.3)	1.52, 1.41–1.64 (6.7/44.5/38.4/10.4)	1.48, 1.36–1.59 (9.8/39.6/43.9/6.7)	0.72
***P*-value**	0.41	**<0.001**	0.11	

Across subspecialties, the addition of clinical presentation to image descriptions significantly improved diagnostic accuracy for several LLMs, most notably Claude 3.5 Sonnet. In gastrointestinal cases, Claude 3.5 Sonnet showed a significant increase in both top 1 accuracy (57.7% [95% CI 38.9–74.5] to 88.5% [95% CI 71.0–96.0], *P* = 0.005) and mean top 3 differential accuracy (1.23 [95% CI 0.92–1.54] to 1.54 [95% CI 1.21–1.87], *P* = 0.034). Claude 3.5 Sonnet also demonstrated a significant improvement in top 1 accuracy in musculoskeletal cases (64.7% [95% CI 47.9–78.5] to 79.4% [95% CI 63.2–89.7], *P* = 0.03) and in genitourinary cases (66.7% [95% CI 45.4–82.8] to 85.7% [95% CI 65.4–95.0], *P* = 0.04). Of note, Gemini 1.5 Pro performed worse in musculoskeletal cases for top 1 accuracy when clinical presentation was added (73.5% [95% CI 56.9–85.4] to 61.8% [95% CI 45.0–76.1], *P* = 0.04). In brain and spine cases, improvements in top 1 accuracy were significant (55.3% [95% CI 39.7–69.9] to 65.8% [95% CI 72.7–94.2], *P* = 0.04). ChatGPT-4 V showed no statistically significant changes in any category. See Appendix Tables 1, 2 and 3 for details.

## Discussion

We evaluated the diagnostic accuracy of three large language models (LLMs) – ChatGPT-4 V, Claude 3.5 Sonnet, and Gemini 1.5 Pro – in generating differential diagnoses for pediatric radiological cases presented in textual format, with and without accompanying clinical history. Our findings demonstrated that all three models exhibited comparable top 1 accuracy when only image descriptions were provided. Adding clinical presentation significantly improved diagnostic accuracy for ChatGPT-4 V and Claude 3.5 Sonnet but not for Gemini 1.5 Pro. There was no statistical difference between LLMs in top 3 differential accuracy in cases with only image description cases and when clinical presentation was added. These results suggest that while LLMs can perform reasonably well based solely on radiological findings, integrating clinical context may enhance diagnostic performance for certain models.

Our research contributes to the expanding body of work investigating the performance of LLMs in radiological diagnostics. Our study shows that LLMs have similar performance in pediatric cases as prior research, which used primarily adult cases. None of the models, aside from Claude 3.5 Sonnet (when clinical presentation was included), exceeded 65% diagnostic accuracy. This is a trend that aligns with previous studies assessing the potential utility of LLMs in medical diagnoses. For example, a study examining LLM performance on 60 neuroradiology quiz cases (based on textual radiological findings alone) found that ChatGPT-4 provided correct differential diagnoses in 61.7% of cases [9]. Similarly, in another study, researchers tested open-source LLMs on 1,933 diverse radiological cases across different radiological subspecialties from the EuroRad library, prompting models with on clinical history and textual descriptions of imaging findings. While Claude and Gemini were not used in this study, ChatGPT-4o had a diagnostic accuracy of 79.6% which were higher than Meta’s Llama-3-70B (73.2) [10]. Another study that investigated the diagnostic capabilities of GPT-4 V and Gemini Vision Pro in solving 190 radiological quiz cases by providing only textual image findings and clinical presentation showed that both models exhibited an accuracy range of 41–49% [6].

Claude 3.5 Sonnet’s superior performance when clinical information presentation was included alongside image descriptions was also consistent with previous research. A study evaluated Claude 3.5’s performance on 322 radiological quizzes by comparing clinical history alone and clinical history and image findings [11]. They discovered a substantial increase in diagnostic accuracy when contextual data was incorporated, with accuracy rates rising from 21.4% to 64.9%. Furthermore another study found that informing Claude about clinical context improved its diagnostic accuracy by approximately 5.3%, suggesting that Claude may apply Bayesian-like reasoning when synthesizing data [12]. The consistency of these findings across multiple studies underscores Claude’s advanced contextual reasoning capabilities and its potential as a valuable tool in radiological diagnostics when provided with comprehensive case details. The study demonstrates the critical importance of incorporating clinical information alongside image descriptions in pediatric imaging when using large language models. While the models showed similar performance based on image descriptions alone, the inclusion of clinical data significantly enhanced diagnostic accuracy for Claude 3.5 Sonnet. This underscores the necessity of integrating comprehensive clinical context to optimize the diagnostic potential of AI in pediatric radiology. Of note, Gemini 1.5 Pro performed significantly worse in musculoskeletal cases for top 1 accuracy when clinical presentation was added while the sample size was small for comparison.

Several limitations should be considered when viewing this study. First, while substantial, the sample size of 164 cases may not be large enough to capture the full spectrum of pediatric radiological presentations. This potentially limits the generalizability of our findings. Second, the case examples were obtained from a pediatric radiology textbook series and thus may not accurately represent real-world clinical presentations across different populations. It is important to note that none of the LLMs had adequate diagnostic accuracy despite the textbook selecting cases with unique radiographic features. Also, case example difficulty may not have been consistent. Future studies can address these limitations by incorporating larger, more diverse datasets. Fourth, the training data for these algorithms have not been fully released to the public, so it is unclear whether the radiology textbook used as the case source in this study was included in the training of LLMs used in this study. Finally, radiological diagnostic accuracy cannot be derived solely from interpreting textual description given that imaging is the most important data in radiology. In a recent study, most LLMs show similar accuracy between text only input and text with image input on analyzing *New England Journal of Medicine* Image Challenge Cases [13]. Notably, this study was conducted when image analysis capability was not available. Future studies will benefit from comparing human radiologist and LLMs on whether inputting images affect diagnostic accuracy and creating differential diagnosis in pediatric cases.

## Conclusion

Our study compared the abilities of ChatGPT-4 V, Claude 3.5 Sonnet, and Gemini 1.5 Pro to generate accurate differential diagnoses for textbook pediatric radiological cases without imaging across a broad range of specialties, finding that all three models produced comparable results with image descriptions alone, while Claude 3.5 Sonnet performed best when both clinical data and image descriptions were inputted. Overall, our research suggests the inclusion of clinical information enhances the diagnostic outcomes and potential of AI, though further research is needed before larger-scale integration of the technology.

## Supplementary Information

Below is the link to the electronic supplementary material.

Supplementary file1 (DOCX 47.7 KB)

## Data Availability

Data is provided within the manuscript or supplementary information files.
