# Supplementary material for: Accuracy of large language models in generating differential diagnosis from clinical presentation and imaging findings in pediatric cases
Source: Pediatr Radiol. 2025 Jul 12;55(9):1927–33. doi: 10.1007/s00247-025-06317-z (PMC12394349; doi:10.1007/s00247-025-06317-z)
Supplement: Supplementary file 1 — Supplementary file1 (DOCX 47.7 KB) [file 247_2025_6317_MOESM1_ESM.docx]

**Appendix Table 1.  Sample Case and Generated LLM Responses**

1. **Cardiac case**

| **Cases** | |  | **LLM Response** | | | | | **Textbook** |
| --- | --- | --- | --- | --- | --- | --- | --- | --- |
| **Image Description** | **Image Description and Clinical Presentation** | **Prompt Format** | | | **ChatGPT-4V** | **Claude 3.5 Sonnet** | **Gemini 1.5 Pro** |  |
| Axial T1-W MRI shows a right anterior chest wall mass that is isointense to muscle and that markedly displaces the trachea to the left. Axial T2-W image shows the mass is hyperintense and multiseptated. Coronal contrast-enhanced T1-W MRI demonstrates a soft-tissue mass with septal enhancement; it extends from the upper chest along the right side of the neck right side of the neck. | In a 16-year-old boy with a chest wall mass, axial T1-W MRI shows a right anterior chest wall mass that is isointense to muscle and that markedly displaces the trachea to the left. Axial T2-W image shows the mass is hyperintense and multiseptated. Coronal contrast-enhanced T1-W MRI demonstrates a soft-tissue mass with septal enhancement; it extends from the upper chest along the right side of the neck right side of the neck. | Image Description Only | | Main Diagnosis | Neuroblastoma | Lymphangioma | Lymphatic Malformation | Lymphatic malformation. |
|  |  |  |  | Top 3 Differential Diagnoses | Neuroblastoma, Rhabdomyosarcoma, pleuropulmonary blastoma | Lymphangioma, Neuroblastoma, Rhabdomyosarcoma | Lymphatic Malformation, Venous Malformation, Neuroblastoma | Lymphatic malformation, Venous malformation,  Necrotic adenopathy |
|  |  | Image Description and Clinical Presentation | | Main Diagnosis | Synovial Sarcoma | Lymphangioma | Lymphoma | Lymphatic malformation. |
|  |  |  |  | Top 3 Differential Diagnoses | Neuroblastoma, Synovial sarcoma, Rhabdomyosarcoma | Lymphangioma, Teratoma, Soft tissue sarcoma | Lymphoma,  germ cell tumor, neurogenic tumor | Lymphatic malformation, Venous malformation,  Necrotic adenopathy |

1. **Genitourinary case**

| **Cases** | |  | **LLM Response** | | | | | **Textbook** |
| --- | --- | --- | --- | --- | --- | --- | --- | --- |
| **Image Description** | **Image Description and Clinical Presentation** | **Prompt Format** | | | **ChatGPT-4V** | **Claude 3.5 Sonnet** | **Gemini 1.5 Pro** |  |
| In a pediatric patient, longitudinal and transverse US of the right upper quadrant shows a solid mass with the suggestion of concentric rings of alternating increased and decreased echogenicity, adjacent to a normal adrenal. Coronal reformatted contrast-enhanced CT shows the mass is partly surrounded by claws of renal tissue, indicating it arose from the kidney. Axial contrast-enhanced CT shows the mass is well circumscribed, compressing a thin rim of renal tissue. | In a 2-month-old with a palpable abdominal mass, longitudinal and transverse US of the right upper quadrant shows a solid mass with the suggestion of concentric rings of alternating increased and decreased echogenicity, adjacent to a normal adrenal. Coronal reformatted contrast-enhanced CT shows the mass is partly surrounded by claws of renal tissue, indicating it arose from the kidney. Axial contrast-enhanced CT shows the mass is well circumscribed, compressing a thin rim of renal tissue. | Image Description Only | | Main Diagnosis | Nephroblastoma | Wilms tumor (nephroblastoma) | Wilms tumor | Mesoplastic c nephroma |
|  |  |  |  | Top 3 Differential Diagnoses | Nephroblastoma, neuroblastoma, multilocular cystic nephroma | Wilms tumor, Mesoblastic nephroma, Clear cell sarcoma of the kidney | Wilms tumor (nephroblastoma), clear cell sarcoma of the kidney, neuroblastoma | Mesoplastic nephroma, wilms tumor, rhabdoid tumor |
|  |  | Image Description and Clinical Presentation | | Main Diagnosis | Congenital mesoblastic nephroma | Congenital Mesoblastic Nephroma (CMN) | Wilms tumor | Mesoblastic nephroma |
|  |  |  |  | Top 3 Differential Diagnoses | Congenital mesoblastic nephroma, wilms tumor, neuroblastoma | Congenital Mesoblastic Nephroma (CMN), Wilms Tumor, Clear Cell Sarcoma of the Kidney (CCSK) | Wilms tumor, Congenital mesoblastic nephroma, Renal cell carcinoma | Mesoplastic nephroma, wilms tumor, rhabdoid tumor |

1. **Head and Neck Case**

| **Cases** | |  | **LLM Response** | | | | | **Textbook** |
| --- | --- | --- | --- | --- | --- | --- | --- | --- |
| **Image Description** | **Image Description and Clinical Presentation** | **Prompt Format** | | | **ChatGPT-4V** | **Claude 3.5 Sonnet** | **Gemini 1.5 Pro** |  |
| In a pediatric patient, axial T2-W MRI shows bilateral intraocular masses that are slightly hypointense to gray matter. T1-W fat-suppressed gadolinium enhanced axial image shows the masses enhance | In an 8-year-old with leukocoria, axial T2-W MRI shows bilateral intraocular masses that are slightly hypointense to gray matter. T1-W fat-suppressed gadolinium enhanced axial image shows the masses enhance | Image Description Only | | Main Diagnosis | Retinoblastoma | Retinoblastoma | Retinoblastoma | Retinoblastoma |
|  |  |  |  | Top 3 Differential Diagnoses | Retinoblastoma, persistent fetal vasculature, coats disease | Retinoblastoma, Coats' Disease, Persistent Hyperplastic Primary Vitreous (PHPV) | Retinoblastoma, Persistent Hyperplastic Primary Vitreous, Coats Disease | Retinoblastoma, persistent fetal vasculature, coats disease |
|  |  | Image Description and Clinical Presentation | | Main Diagnosis | Retinoblastoma | Retinoblastoma | Retinoblastoma | Retinoblastoma |
|  |  |  |  | Top 3 Differential Diagnoses | Retinoblastoma, coats disease, persistent fetal vasculature | Retinoblastoma, Coats' disease, Persistent hyperplastic primary vitreous | Retinoblastoma, Diffuse uveal melanoma, Lymphoma | Retinoblastoma, persistent fetal vasculature, coats disease |

**Appendix Table 2.  Comparison of Top 1 Accuracy between ChatGPT-4V, Claude 3.5 Sonnet, and Gemini 1.5 Pro in different radiological subspecialties**

| **Comparison of Prompt Formats Within LLMs** | | | | | | | |
| --- | --- | --- | --- | --- | --- | --- | --- |
|  | ChatGPT-4V | Claude 3.5 Sonnet | | Gemini 1.5 Pro | | P value | |
|  | %, 95% CI  (#/total) | %, 95% CI  (#/total) | | %, 95% CI  (#/total) | |  | |
| ***Cardiac (N=27)*** | | |  | |  | |  |
| Image Description Only | | |  | |  | |  |
|  | 44.4, 27.6-62.7  (12/27) | 51.9, 34.0-69.3  (14/27) | | 55.6, 37.3-72.4  (15/27) | | 0.58 | |
| Image Description With Clinical Presentation | | | |  | | | |
|  | 51.9, 34.0-69.3  (14/27) | 66.7, 47.8-81.4  (18/27) | | 59.3, 40.7-75.5  (16/27) | | 0.45 | |
| **P value** | 0.32 | 0.21 | | 0.74 | |  | |
| ***Gastrointestinal (N=26)*** | | | | | | | |
| Image Description Only | | | | | | | |
|  | 38.5, 22.4-57.5  (10/26) | 57.7, 38.9-74.5  (15/26) | | 46.2, 28.8-64.5  (12/26) | | 0.15 | |
| Image Description With Clinical Presentation | | | | | | | |
|  | 53.8, 35.5-71.2  (14/26) | 88.5, 71.0-96.0  (23/26) | | 61.5, 42.5-77.6  (16/26) | | **<0.001** | |
| **P value** | 0.10 | **0.005** | | 0.10 | |  | |
| ***Genitourinary (N=21)*** | | | | | | | |
| Image Description Only | | | | | | | |
|  | 57.1, 36.5-75.5  (12/21) | 66.7, 45.4-82.8  (14/21) | | 57.1, 36.5-75.5  (12/21) | | 0.61 | |
| Image Description With Clinical Presentation | | | | | | | |
|  | 71.4, 50.0-86.2  (15/21) | 85.7, 65.4-95.0 (18/21) | | 71.4, 50.0-86.2 (15/21) | | 0.37 | |
| **P value** | 0.18 | **0.04** | | 0.18 | |  | |
| ***Musculoskeletal (N=34)*** | | | | | | | |
| Image Description Only | | | | | | | |
|  | 58.8, 42.2-73.6  (20/34) | 64.7, 47.9-78.5  (22/34) | | 73.5, 56.9-85.4  (25/34) | | 0.15 | |
| Image Description With Clinical Presentation | | | | | | | |
|  | 61.8, 45.0-76.1 (21/34) | 79.4, 63.2-89.7 (27/34) | | 61.8, 45.0-76.1 (21/34) | | **0.03** | |
| **P value** | 0.75 | **0.03** | | **0.04** | |  | |
| ***Head and Neck (N=18)*** | | | | | | | |
| Image Description Only | | | | | | | |
|  | 55.6, 33.7-75.4  (10/18) | 61.1, 38.6-79.7  (11/18) | | 88.9, 67.2-96.9  (16/18) | | **0.02** | |
| Image Description With Clinical Presentation | | | | | | | |
|  | 72.2, 49.1-87.5 (13/18) | 72.2, 49.1-87.5 (13/18) | | 72.2, 49.1-87.5 (13/18) | | 1.0 | |
| **P value** | 0.18 | 0.41 | | 0.18 | |  | |
| ***Brain and Spine (N=38)*** | | | | | | | |
| Image Description Only | | | | | | | |
|  | 73.7, 58.0-85.0  (28/38) | 78.9, 63.7-88.9  (30/38) | | 55.3, 39.7-69.9  (21/38) | | **0.02** | |
| Image Description With Clinical Presentation | | | | | | | |
|  | 73.7, 58.0-85.0 (28/38) | 86.8, 72.7-94.2 (33/38) | | 65.8, 49.9-78.8 (25/38) | | **0.05** | |
| **P value** | 1.0 | 0.18 | | **0.04** | |  | |

**Appendix Table 3.  Comparison of Top 3 Differential Accuracy between ChatGPT-4V, Claude 3.5 Sonnet, and Gemini 1.5 Pro in different radiological subspecialties**

| **Comparison of Prompt Formats Within LLMs** | | | | | | | |
| --- | --- | --- | --- | --- | --- | --- | --- |
|  | ChatGPT-4V | Claude 3.5 Sonnet | | Gemini 1.5 Pro | | P value | |
|  | Mean Score, 95% CI  Score Distribution (0/1/2/3) % | Mean Score, 95% CI  Score Distribution (0/1/2/3) % | | Mean Score, 95% CI  Score Distribution (0/1/2/3) % | |  | |
| ***Cardiac (N=27)*** | | |  | |  | |  |
| Image Description Only | | |  | |  | |  |
|  | 1.00, 0.60-1.40  (40.7/25.9/25.9/7.4) | 1.22, 0.84-1.61 (29.6/25.9/37.0/7.4) | | 1.11, 0.76-1.46 (25.9/44.4/22.2/7.4) | | 0.63 | |
| Image Description With Clinical Presentation | | | |  | | | |
|  | 1.19, 0.77-1.60 (33.3/25.9/29.6/11.1) | 1.63, 1.28-1.98 (11.1/29.6/44.4/14.8) | | 1.44, 1.08-1.81  (18.5/29.6/40.7/11.1) | | 0.06 | |
| **P value** | 0.27 | **0.02** | | 0.13 | |  | |
| ***Gastrointestinal (N=26)*** | | | | | | | |
| Image Description Only | | | | | | | |
|  | 0.88, 0.58-1.19 (34.6/42.3/23.1/0.0) | 1.23, 0.92-1.54 (11.5/61.5/19.2/7.7) | | 1.38, 1.08-1.69 (15.4/30.8/53.8/0.0) | | **0.008** | |
| Image Description With Clinical Presentation | | | | | | | |
|  | 1.12, 0.75-1.48 (26.9/42.3/23.1/7.7) | 1.54, 1.21-1.87 (3.8/53.8/26.9/15.4) | | 1.69, 1.44-1.94  (3.8/26.9/65.4/3.8) | | **0.006** | |
| **P value** | 0.09 | **0.03** | | 0.06 | |  | |
| ***Genitourinary (N=21)*** | | | | | | | |
| Image Description Only | | | | | | | |
|  | 1.48, 1.08-1.87 (14.3/33.3/42.9/9.5) | 1.38, 1.01-1.75 (14.3/38.1/42.9/4.8) | | 1.10, 0.75-1.45 (23.8/42.9/33.3/0.0) | | 0.06 | |
| Image Description With Clinical Presentation | | | | | | | |
|  | 1.33, 0.94-1.72 (19.0/33.3/42.9/4.8) | 1.48, 1.20-1.75 (4.8/42.9/52.4/0.0) | | 1.38, 0.99-1.77  (19.0/28.6/47.6/4.8) | | 0.56 | |
| **P value** | 0.26 | 0.53 | | 0.11 | |  | |
| ***Musculoskeletal (N=34)*** | | | | | | | |
| Image Description Only | | | | | | | |
|  | 1.26, 1.02-1.51 (8.8/61.8/23.5/5.9) | 1.47, 1.22-1.72 (2.9/55.9/32.4/8.8) | | 1.59, 1.33-1.85 (5.9/38.2/47.1/8.8) | | 0.08 | |
| Image Description With Clinical Presentation | | | | | | | |
|  | 1.32, 1.07-1.58 (8.8/55.9/29.4/5.9) | 1.41, 1.15-1.67 (5.9/55.9/29.4/8.8) | | 1.59, 1.33-1.85  (2.9/47.1/38.2/11.8) | | 0.20 | |
| **P value** | 0.53 | 0.41 | | 0.98 | |  | |
| ***Head and Neck (N=18)*** | | | | | | | |
| Image Description Only | | | | | | | |
|  | 1.56, 1.17-1.95 (0.0/61.1/22.2/16.7) | 1.39, 0.97-1.81 (11.1/50.0/27.8/11.1) | | 1.67, 1.33-2.01 (5.6/27.8/61.1/5.6) | | 0.50 | |
| Image Description With Clinical Presentation | | | | | | | |
|  | 1.44, 1.14-1.75 (0.0/61.1/33.3/5.6) | 1.50, 1.11-1.89 (5.6/50.0/33.3/11.1) | | 1.33, 0.95-1.71  (11.1/50.0/33.3/5.6) | | 0.73 | |
| **P value** | 0.52 | 0.48 | | 0.08 | |  | |
| ***Neuro (N=38)*** | | | | | | | |
| Image Description Only | | | | | | | |
|  | 1.55, 1.33-1.78 (5.3/39.5/50.0/5.3) | 1.39, 1.12-1.67 (13.2/42.1/36.8/7.9) | | 1.37, 1.15-1.59  (5.3/57.9/31.6/5.3) | | 0.31 | |
| Image Description With Clinical Presentation | | | | | | | |
|  | 1.53, 1.30-1.75 (2.6/50.0/39.5/7.9) | 1.58, 1.32-1.84 (7.9/36.8/44.7/10.5) | | 1.37, 1.15-1.59 (7.9/50.0/39.5/2.6) | | 0.16 | |
| **P value** | 0.76 | 0.09 | | 1.0 | |  | |
